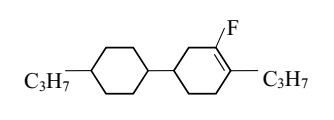# SPR Effect Controlled by an Electric Field in a Tapered Optical Fiber Surrounded by a Low Refractive Index Nematic Liquid Crystal

**DOI:** 10.3390/ma13214942

**Published:** 2020-11-03

**Authors:** Joanna Korec, Karol A. Stasiewicz, Leszek R. Jaroszewicz, Katarzyna Garbat

**Affiliations:** 1 Institute of Applied Physics, Military University of Technology, 00-908 Warsaw, Poland; karol.stasiewicz@wat.edu.pl (K.A.S.); leszek.jaroszewicz@wat.edu.pl (L.R.J.); 2 Institute of Chemistry, Military University of Technology, 00-908 Warsaw, Poland; katarzyna.garbat@wat.edu.pl

**Keywords:** optical fiber, liquid crystal, surface plasmon resonance, metallic layer, tapered fiber sensor

## Abstract

This paper presents the influence of a thin metal layer deposition on the surface of a tapered optical fiber surrounded by a low liquid crystal, on light propagation inside the taper structure. In this research, three types of liquid crystal cells were under investigation: orthogonal, parallel, and twist. They differed by the rubbing direction of the electrodes in relation to the fiber axis determining the initial molecule arrangement inside the cell. Gold films with thickness d = 30 nm were deposited on the tapered fiber surface in the tapered waist area. Cells including a tapered optical fiber with no metallic layer were also examined and presented as a reference. All measurements were performed at room temperature for a different steering voltage U from 0 to 200 V, with and without any amplitude modulation with a frequency f = 5 Hz, and the wavelength λ range from 550 to 1200 nm. As a result, the resonant peaks were obtained, which depends on a liquid crystal cell type and steering voltage, as well. This paper shows the possibility of sensing the change of applied voltage by the constructed system. During measurements, additional effects as signal overlapping and intermodal interference were observed reducing measured voltage value. In the future, the improved, similar systems that will have a better response could be used as a sensor of factors to which liquid crystal (LC) will be sensitive, especially temperature and electric field.

## 1. Introduction

Phenomenon of a surface plasmon resonance (SPR) is commonly used in the sensing techniques because of its simplicity and very high accuracy [1]. There are few possible configurations where SPR can be easily observed, e.g., a prism with a metallic layer in the Kretschmann [2] and Otto [3] configuration, sensors based on Bragg’s grating, optical fiber, and microfiber [4].

**Figure 1:**
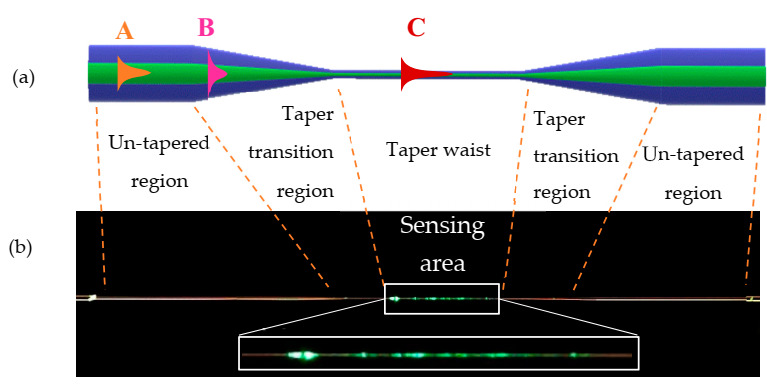
Scheme of (**a**) light propagation in different sections of the tapered fiber and (**b**) photo of a tapered optical fiber with a visible evanescent field.

Optical fiber taper manufacturing is based on heating the fiber to its softening temperature (about 1100–1200 °C) and elongating it to an appropriate length during the tapering process [5]. Reducing the core-cladding diameter is achieved by pulling on both ends of the fiber in opposite directions under the burner flame. Figure 1a presents the scheme of the tapered optical fiber where its three sections are presented. The first one is a section of an untapered optical fiber, then the region with a diameter gradual change and, subsequently, the region that shows a quite uniform diameter and the same on a reverse order. The geometry of these parts has a significant influence on the light propagation in the tapered fiber. The rate of taper’s diameter change is one of the most important criteria in the manufacturing of good-quality tapered optical fibers. In the case of some tapered fibers, when the fundamental mode couples to the higher-order modes, the losses of the optical power may occur. It is related to the energy flow from higher-order modes, which recombine and interfere with fundamental mode and makes this effect highly undesirable [5,6].

According to Figure 1a, part A corresponds with the fiber untapered part where light is propagating only in the core of the fiber, however, some of the incident energy penetrates the cladding as an evanescent wave (EW) [7,8]. In the standard single-mode optical fiber this wave is typically very low at interface core-cladding and there it is not possible for EW to interact with an external medium [8]. At part B, where the diameter is gradually reduced, the wave starts penetrating the cladding. Finally, at part C the wave propagates in the whole structure of the tapered waist and leaks out to the external medium. Only in this part the waist became the new core and surrounding medium, the cladding, hence the EW can interact with an external medium [8,9].

During the elongation of the optical fiber and the creation of the taper, the radius of the core changes, and its effective refractive index, hence the geometry of these parts has a significant influence on the light propagation inside the tapered fiber. This results in widening of the beam and changing the normalized frequency [10]. A large difference of the refractive index between air and optical fiber supports more than one mode, hence the tapered region is converted into a multimode fiber. Due to this specific property and the increasing of the participation of light, which propagates as an evanescent field, it makes this region suitable for sensing purposes. The efficient interaction depends on the penetration depth *d_p_*, which is a distance at which the electric field penetrates the second medium and decreases to 1/*e* of its initial value at the surface and is given as [11]:

(1) $$d_{p}=\frac{\lambda }{2\pi \sqrt{n_{core}^{2}sin^{2}\theta_{i}-n_{enviroment}^{2}}}$$

where:

*λ*—wavelength of the incident light,
*n_core_*, *n_enviroment_*—refractive index of the new core and the external environment (dielectric medium),
*θ_i_*—incident angle of a wave.

### Physics of Surface Plasmon Resonance

Surface plasmon resonance is a well-known phenomenon used in sensing techniques. The metal–dielectric interface due to high charge density fluctuations generates an electromagnetic excitation called surface plasmons. The quantum of these oscillations is called surface plasmons and is accompanied by transverse magnetically (TM) polarized waves [12,13]. The electric field penetrates both media (metal and dielectric) and decays exponentially wherein the maximum of this field occurs at the metal–dielectric interface [13,14]. This specific characteristic of surface plasmons can be found by solving Maxwell’s equation for semi-infinitive media with interface metal–dielectric. [15]. Surface plasmons are p-polarized waves, so these can be exited only by TM waves. The phenomenon of the resonance between these waves occurs when their wave vectors are the same. It results in the energy transfer from the incident wave to the surface plasmon wave (SPW) [15]. The propagation constant of SPW is given by:

(2) $$K_{SP}=\;\frac{\omega }{c}\sqrt{\frac{\varepsilon_{m}\varepsilon_{s}}{\varepsilon_{m}+\varepsilon_{s}}}$$

where:

*ε_m_*, *ε_s_*—dielectric constants of metal and dielectric medium,
*ω*, *c*—frequency and light velocity in the vacuum, respectively.

The propagation constant of the light wave that propagates through a dielectric medium is given by:

(3) $$K_{S}=\;\frac{\omega }{c}\sqrt{\varepsilon_{s}}$$

The comparison of the above equations implies that *K_SP_* > *K_S_*, for the dielectric constant of metal *ε_m_* < 0, and dielectric *ε_s_* > 0. However, as it was said before, both wave vectors must be equal for excitation of surface plasmon. Summarizing this inequality, the direct light cannot excite the surface plasmon, but excitation can be achieved if the wave vector of light in the dielectric medium is increased. It is possible by using EW instead of a direct light [15,16]. The propagation constant of the EW is given by:

(4) $$K_{EW}=\;\frac{\omega }{c}\sqrt{\varepsilon_{F}}\;sin\theta$$

where:

*K_EW_*—the propagation constant of EW,
$\varepsilon_{F}$—dielectric constant of the fiber material,
*θ*—incident angle of a TM wave.

The wave vector of SPW depends on the surrounding medium properties, especially on its dielectric constant. Any change in the medium dielectric constant causes changes in the wave vector of the SPW, and a change in the wave vector of the incident light at resonance [17,18,19]. Typically, the SPR sensors use a prism covered with the metal layer, where the EW is generated when the angle of incidence is greater than the critical one. In this configuration, the angular measurements were performed. However, in the optical fiber taper, the light beam propagated due to a total internal reflection (TIR) and there was no possibility to change the light beam angle of incidence, so instead of angular, the spectral method was used [20]. The optical fiber-based sensor contains liquid crystal (LC) as a surrounding medium and the tapered optical fiber with a deposited metallic layer in the tapered waist area. Achieved resonant dip, the occurring wavelength, its width, and depth depends on the refractive index of the surrounding medium, type, and quality of the deposited metal [21].

**Figure 2:**
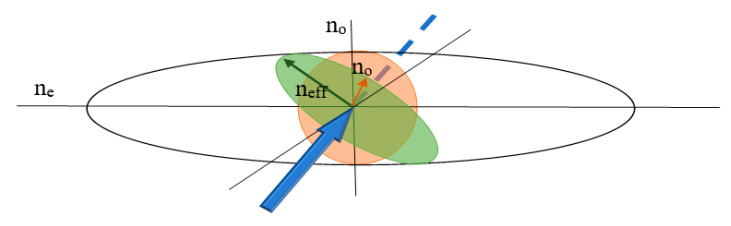
The scheme of an optic axis where birefringence is equal to ne-no and is a function of the applied voltage. Any impinging beam splits into an ordinary and an effective refractive index *n*_eff_.

The use of LC (as an external medium) possesses anisotropic properties and by the application of an electric field, it is possible to change the molecules orientation and, eventually, influence on the light transmission [22,23]. As it is presented in Figure 2, the liquid crystal (LC) molecules possess two different refractive indices: ordinary (*n*_o_) and extraordinary (*n*_e_), in relation to the measuring axis. However, there is also the effective refractive index (*n*_eff_), which is between them and in view of this research has the highest importance because it can be easily changed by thermal, electric, and magnetic fields [24].

**Figure 3:**
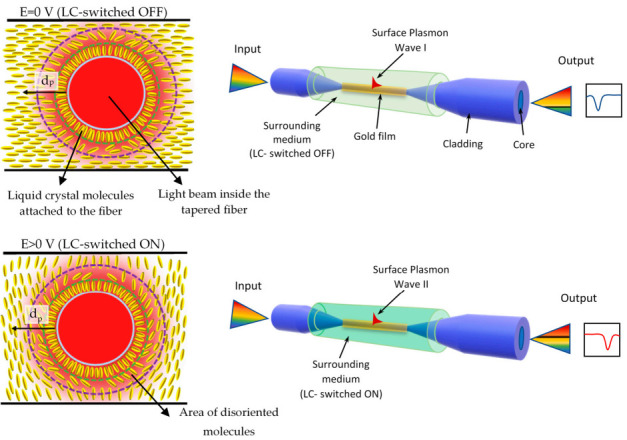
Surface plasmon resonance (SPR) effect in the optical fiber taper-based sensor.

Generally, a different refractive index of a waveguide cladding (LC around the tapered fiber) depends on the orientation of *n*-director molecules in the external electric field. Any changes that influence LC may be recorded as a change in transmission or in phase shift. Around the taper, an area of disoriented LC molecules is formed attached to the fiber [25]. Present LC cell manufacturing technique does not allow one to decrease this phenomenon, however, this issue will be taken under investigation in further research. Figure 3 presents the SPR effect in the optical fiber taper-based sensor with LC cladding.

The recent thin film deposition techniques make it possible to modify fiber surfaces with nano-coating layers. This allows one to improve the sensors based on the phenomena of SPR, localized SPR, and lossy mode resonance to detect changes of the surrounding medium by measuring the spectral shift of the plasmonic [25,26,27]. Basing on this information, it can be stated that the connection of a tapered optical fiber covered with a metal layer with the external material, which possesses variable parameters (LC) will allow one to combine two effects: SPR effect caused by the metal layer and the effect caused by changing the effective refractive index of LC around the taper. Additionally, there is some study on a connection of LC with metallic layers, but performed using the glass prism [28,29].

## 2. Materials and Methods

### 2.1. Preparation of Liquid Crystal Cells

Tapered optical fibers were made from the optical fiber SMF @ 1550 using the fiber optic taper element technology (FOTET–Fiber Optic Taper Element Technology) system. Scheme and a detailed description of the manufacturing process are included in the previous articles, hence it will not be presented in this paper [30]. Obtained tapered fibers were characterized by the following parameters: length of the tapered waist L = 20.20 ± 0.12 mm, diameter of the taper waist Φ = 15.5 ± 0.5 μm, and loss a = 0.20 ± 0.04 dB. The structure was secure by using a special frame designed in the Autocad software and printed by a 3D printer. Dimensions of the frame were chosen for the initial protection of the tapered fiber and enabled a uniform deposition of metallic layer, and the frame, allowing further assembling of the liquid crystal cell. The gold layers were sputtered by Leica sputter coater EM SCD005 equipped with a quartz crystal microbalance (QCM) sensor. Coating processes were carried out in a flow of an argon, chamber pressure *p* = 10^−2^ mbar and current I = 30 mA.

**Figure 4:**
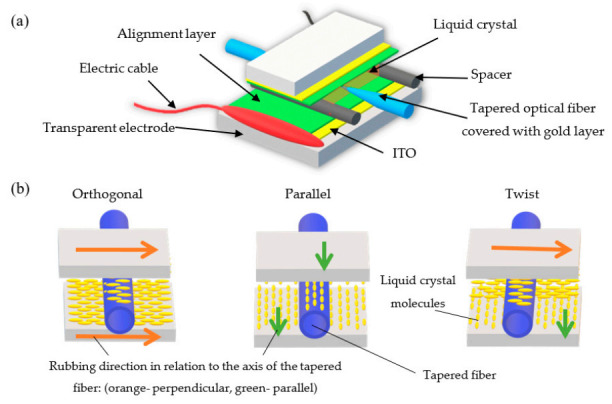
Scheme of the: (**a**) liquid crystal (LC) cell and (**b**) types of the investigated cells: orthogonal, parallel, and twist.

For the purpose of this research, the gold layers thickness was equal to d = 30.0 ± 0.5 nm. In the next stage of preparation, the covered tapers were closed between two transparent electrodes into “sandwich” type cells. Each electrode was covered with indium tin oxide (ITO) and an alignment layer, which provides both good current conduction on the surface of the electrode and initial orientation of the molecules in the cell. The width of the electrodes was equal to w = 5 mm, which corresponds to the width of the tapered waist and sensor area, as well. To maintain the constant distance between the electrodes, the spacers were used, in this research, the spacers had the diameter d_s_ = 40 μm. This dimension allows for an easy placement of the tapered fiber between electrodes and, also, the electric filed needed to steer LC cell can be simultaneously decreased. The scheme of the LC cell is presented in Figure 4a.

In this research, the three types of LC cells, previously described also in [30], were under investigation. They were different from each other in terms of the rubbing direction of the alignment layer on the electrodes in relation to the optical fiber axis. Rubbing direction has a significant influence on the initial arrangement of the LC molecules inside the cell and allows one to obtain a different effective RI around the tapered optical fiber, hence orthogonal, parallel, and twist cells were manufactured. According to Figure 4b, the first type of the cell was an orthogonal cell, which includes electrodes with the same rubbing direction, perpendicular to the axis of the fiber. The parallel cell also had both of the same electrodes, however, their rubbing direction was parallel to the optical fiber. The last type of cell was a twisted cell in which the upper electrode had a perpendicular rubbing direction and the bottom electrode had a parallel rubbing. This configuration allowed us to obtain a twisted nematic effect around the tapered fiber.

### 2.2. Materials

The cells were filled with a nematic liquid crystal mixture donated with 3092 A. Chosen LC was characterized by a low birefringence, simultaneously possessed ordinary refractive index (*n*_o_) lower than refractive index of the tapered optical fiber (*n*_T_). Additionally, 3092 A had a positive dielectric anisotropy, which means the director (vector of the average arrangement of molecules in the LC cell) affected by voltage was parallel to the electric field lines. Composition and basic properties of the 3092 A LC are presented in Table 1.

### 2.3. Experimental

**Figure 5:**
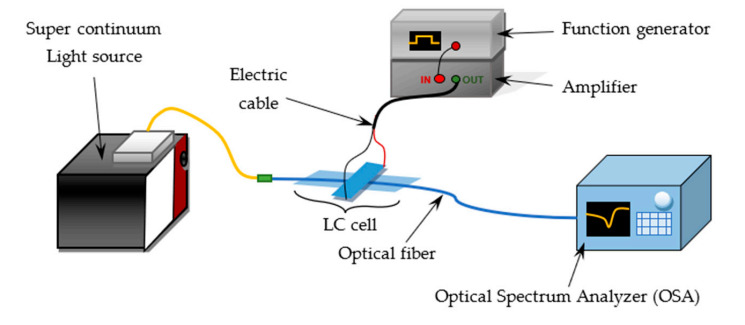
Scheme of the measuring system.

In this research, influence of the metallic layer deposited on the optical fiber taper on the light propagation in the LC cell was under investigation. To achieve spectral characteristics, the measurement system was built of a broad-spectrum light source-SuperK EXTREME supercontinuum (SC), from NKT Photonics (Birkerød, Denmark), and Optical Spectrum Analyzer (OSA) Yokogawa AQ6373B (Japan, Tokyo) with the ability to provide short time and accurate analysis of the short wavelength range between 350 and 1200 nm used for the light analysis. Electric signal necessary to the steering LC cell was generated by the function generator RIGOL DG1022Z (Beijing, China). The spectral analysis was conducted at room temperature for cells with three various molecules orientations and with and without 30 nm gold layer. In order to investigate the differences between cells, the measurements were performed under the various voltage U in the range of 0–200 V, without modulation of an output signal and with an amplitude modulation (AM) with frequency *f* equal to 5 Hz and a 100% depth. Figure 5 presents the used measuring system.

## 3. Results

**Figure 6:**
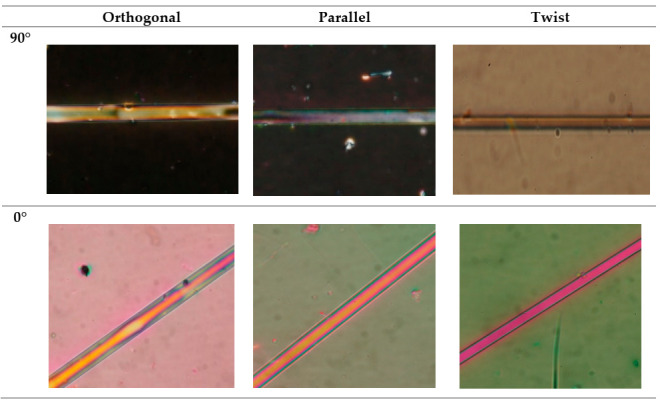
Photos of LC cells: orthogonal, parallel, and twist for crossed (90°) and parallel (0°) polarizers.

In Figure 6 photos of orthogonal, parallel, and twist cells performed on the polarizing microscope Quanta 3D FEG Dual Beam [32] are presented. Photos were taken for two different arrangements of two polarizers: cross and parallel (90° and 0°) in relation to each other and there was used the maximum magnification of 50×. In the images one can see the tapered optical fibers and the surrounding LC. In the first photos obtained for crossed polarizers, it can be seen the plain dark field in the case of the orthogonal and parallel cell and the right one in the case of the twisted cell. It indicates the same arrangement on LC molecules in a whole volume of the cell. Simultaneously, in the photos for a parallel polarizer the tapers and their near neighborhood were visible as colorful glows. This phenomenon is related to the formation of a layer of molecules attached to the taper and it commonly occurs on the glass surface due to hydroxyl groups [33]. From a sensory point of view, it is an undesirable effect, because the EW penetration depth is finite and the energy necessary to reorient these molecules is very high.

Liquid crystals due to their chemical structure possess anisotropic physical properties. This characteristic allows one to steer their arrangement (homeotropic or planar) by using for example the electric field. LC as an environment around the tapered fiber waist creates a medium that is characterized by an effective refractive index and interacts with the evanescent field. However, this *n*_eff_ is related to the initial arrangement of molecules inside the cell and can be achieved by a different rubbing of the alignment layer on the electrodes used to manufacture the LC cell [34].

**Figure 7:**
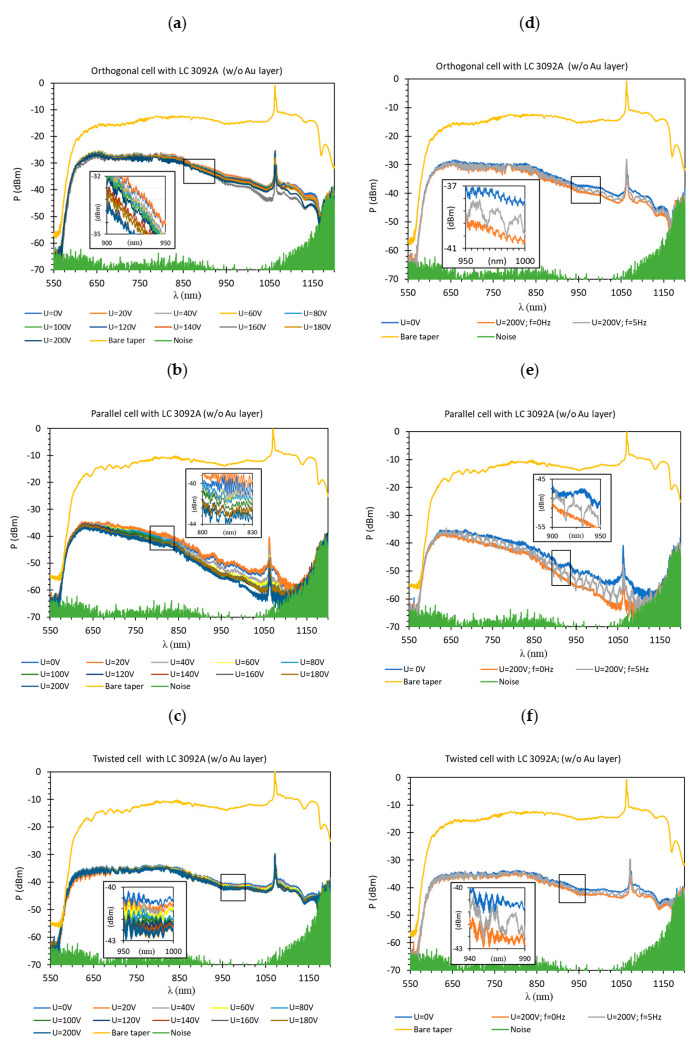
The spectra obtained for the LC cell without metallic layers (**a**) orthogonal; (**b**) parallel; and (**c**) twisted, under steering voltage U in the range of 0–200 V. Spectra obtained for: (**d**) orthogonal; (**e**) parallel; and (**f**) twisted cell for the steering voltage U = 0 and 200 V without and with a 100% depth AM, at frequency f = 5 Hz.

In this paper, at first, three types of LC cells without metallic were examined: orthogonal, parallel, and twist. The results in Figure 7 present the spectral characteristics obtained for LC cells filled with 3092 A without any gold layer and, also, present the influence of the applied steering voltage *U* in the range of 0–200 V. In all graphs, the highest power (yellow color) corresponded to the spectra obtained for a bare optical fiber taper in air and the lowest power (light green color) with the noise level of OSA. Analyzing all the obtained results presented in Figure 7a–c, it can be noticed that an orthogonal cell for the wavelength range of 550–850 nm had the highest power level and it equaled approximately P = −25 dBm, and for the wavelength range of 850–1100 nm slightly decreased from P = −30 dBm to P = −45 dBm (Figure 7a). The very similar characteristics had the twisted cell (Figure 7c), however, its power level for the same wavelength section was lower and equaled P = −35 dBm and P = −40/−45 dBm, for the wavelength λ range of 550–850 nm and 850–1100 nm, respectively. In the case of the parallel cell, this type of LC arrangement caused the highest attenuation. The power level for the wavelength λ range of 550–850 nm varied between −35 and −45 dBm, and for the wavelength λ range of 850–1100 nm between −45 and −62 dBm. However, the lower power level was only slightly above the noise level. Additionally, Figure 7d–f presents the influence of AM on the steering voltage, where the cells dynamic response can be observed. It is assumed that the LC cell is turned OFF when the U = 0 V and is turned ON when U = 200 V. As it can be noticed, when the higher voltage was applied, the lower power was observed.

For all cases, the highest power corresponded to the shorter wavelengths and this effect was a result of different penetration depths of the wavelengths. Since if longer waves penetrate deeper into the surrounding medium, it allows for a better impact of LC, hence the observed power is lower for the waves above 850 nm [35]. When the LC cell is switched off, *n*_eff_ < *n*_T_-the guided power is the highest, however, when the applied voltage is increased, the LC cell is switched on. Theoretical research has proved that the TE (transverse electric) modes penetrate the external medium and TM modes propagate inside the taper. It implies that the TE modes interact with the liquid crystal cladding and are responsible for power change [36,37]. Hence, power decreasing and dynamic switching are the effects of changing in the effective refractive index of LC. Despite the fact that the LC possesses a higher refractive index than the tapered fiber, the wave propagation is still possible, because, for a diameter of the tapers up to 15 μm, the core plays a significant role in the wave propagation [38,39,40]. In addition, the fluctuations visible in the magnification are the modal interference caused by a slight difference between *n*_T_ and LC *n*_eff_.

Additionally, the bare tapered fibers in contrast to the used electrodes did not have the alignment layer, so on their surface were ions OH^−^, and LC molecules were attached to them randomly. The created “coat” of the molecules was characterized by a very high energy, hence the voltage necessary to start reorienting LC was relatively high 20 V.

**Figure 8:**
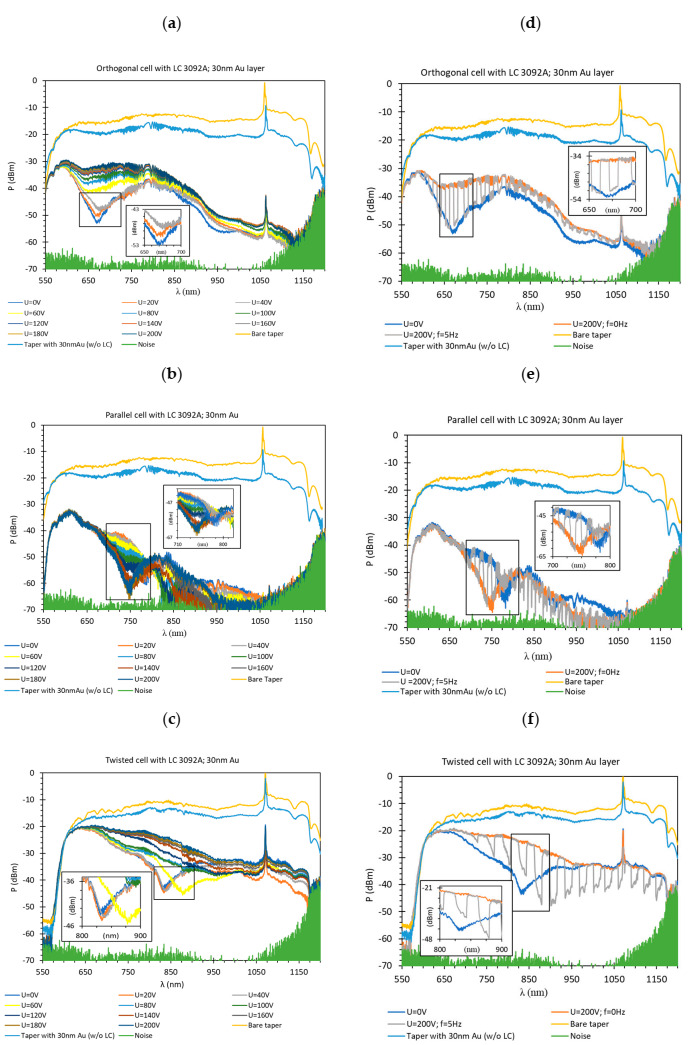
The spectra obtained for a LC cell with deposited 30 nm gold layers (**a**) orthogonal; (**b**) parallel; and (**c**) twisted, under steering voltage U in the range of 0–200 V. Spectra obtained for: (**d**) orthogonal; (**e**) parallel; and (**f**) twisted cell for the steering voltage U = 0 and 200 V without, and with a 100% depth AM, at frequency f = 5 Hz.

In the next stage of measurements, the influence of thin gold layers on the light propagation was examined. Figure 8 presents the obtained results for a 30 nm Au layer and a 3092 A LC mixture. In all graphs beside the spectra obtained for the bare tapered fiber (in yellow), the power levels corresponding to the tapered optical fiber covered with a 30 nm Au measured in the air were introduced (in light blue color). As it can be noticed, the attenuation caused by deposited metal layers (measured in the air) was insignificant and uniform throughout the range. Further filling the LC cell with 3092 A contributed to the appearing of SPR dips, and furthermore, the type of the cell had an impact on the wavelength where these dips occurred. 

Analyzing the spectra in Figure 8a-c it can be observed that for all cases resonant dips occurred without the applied electric field. For the following types of the cells: orthogonal, parallel, and twist the resonant dips were established as 660 nm, 770 nm, and 840 nm, respectively. In the case of the orthogonal and twisted cell, increasing of the steering voltage U in the range of 20–40 V (orthogonal cell) and 20–60 V (twisted cell) caused a slight shift of resonant peaks toward the longer wavelengths, however, a further increase of the steering voltage caused disappearing of these peaks. On this basis, it can be stated that the LC *n*_eff_ below U = 60 V was lower than *n*_T_ and further increasing of steering voltage caused increasing of the effective refractive index higher than the refractive index of the taper [41]. The parallel cell behaved quite differently, applying the electric field did not cause vanishing of resonant dips, but made it deeper and shifted it to the shorter wavelength, also, the power level was slightly the same for all used steering voltages. Probably this was a result of attached molecules parallel to the surface of the fiber. It can be stated that the differences between peaks locations were the effect of the molecules orientation (different *n*_eff_) around the tapered waist. It should be noted that only the TM modes caused the surface plasmon excitation, but an output signal was the result of both propagating modes: TM and TE.

**Figure 9:**
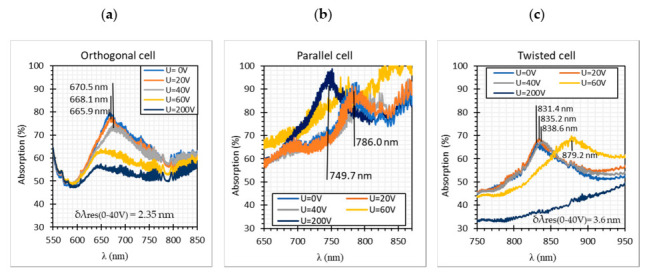
Absorption of SPR dips: (**a**) orthogonal; (**b**) parallel; and (**c**) twisted cell.

Figure 8d–f presents the spectra obtained for U = 0 V and 200 V with AM of the steering voltage, where the cells dynamic response could be observed. What is interesting, for all cases, the signal modulation was visible for the whole spectrum including the resonant dips areas. Additionally, wavelengths in which the LC cells operated were different. Orthogonal and twisted cells were working in the whole measuring spectrum, but the parallel cell had the cut off at 1050 nm when the LC cell was switched off, and at 950 nm when it was switched on. The calculated absorptions for orthogonal, parallel, and twisted are presented in Figure 9.

According to the obtained results, the resonant dip of a parallel cell had the highest absorption, but it can be related to the arrangement of LC molecules because a parallel cell without the metallic layer also had the lowest power level of all. Additionally, the reason why the resonant peaks did not absorb the whole wavelength was that the resonant dip depth highly depended on the thickness of a metallic layer and a quality of the deposited layer including the vacuum level during the metal deposition process, and of a taper ratio and taper profiles [19,42]. In the case of the orthogonal and twisted cells, it was possible to measure shifts of the resonant peaks for the voltage U range of 0–40 V and they were equal to 2.35 nm and 3.60 nm, respectively. Due to the high modal interference for the parallel cell, it was very difficult to measure this shift for the same voltage range.

**Figure 10:**
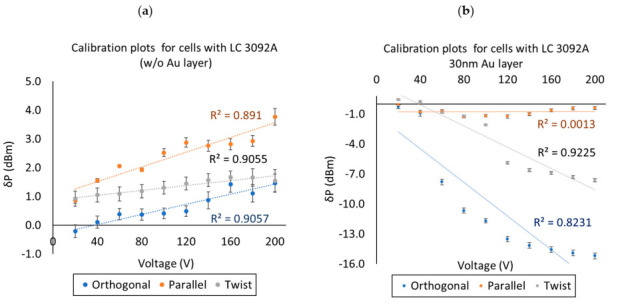
Estimation of the calibration plot for 700 nm. (**a**) cells without gold layer and (**b**) cells with deposited gold layer.

The high intermodal interference was also the main problem for the estimation of the calibration plot (and the determination of standard deviation values). For data presented in Figure 7, Figure 8 and Figure 9 five measurements were performed for each voltages’ value. It can be observed that the difference between the highest and the lowest power changes between 0.023 and 0.275 dBm and depended on the wavelength. For this reason, below in Figure 10 the example of calibration plots for wavelength 700 nm for LCCs with and without Au layer are presented.

## 4. Conclusions

The performed research on the influence of a deposition thin metal layer on the tapered optical fiber surface and closed inside the LC cell allowed for the following conclusions to be drawn:

1. The sensing possibility of constructed system depended on cell type (orthogonal, parallel, and twist), and the used metallic layer.
2. The initial molecules arrangement inside the LC cell had a significant influence on the occurrence and location of the resonant peak. For the orthogonal cell a resonant peak occurred at 660 nm, for the parallel cell at 770 nm and for the twisted cell at 840 nm.
3. The highest resonant peak absorption occurred for the parallel cell and equaled 95% when the cell was switched off and 98% when it was switched on.
4. Dynamic response (switching on/off) visible in the whole spectrum obtained for LC cells with the deposited metallic layer proved that it was possible to modify the light beam propagation properties including the resonant area.
5. The resulting structure can be used in the future as a sensor of factors to which LC were sensitive, especially temperature and electric field. Additionally, it can be a filter for selected wavelengths depending on the type of cell used.

This paper shows a sensing possibility of the constructed system and it should be mentioned that many issues should be improved including the manufacturing technique. The observed effect was quite small and the variations as a function of the applied voltage require further investigation to use these structures in real applications. Additionally, during the measurements, additional effects occurred, e.g., the signal overlapping and intermodal interference, which decreased the level of signal detection. For this reason, the obtained results could be regarded as exploratory work that could lay the foundations for the study of real, optimized systems and might be used in practice.

## Figures and Tables

**Table 1:** Properties of the LC mixture 3092 A [31].

No	Substrates		Properties	
	R1	R2		
Ia	C_3_H_7_	CH_3_	cl. p (°C)	63.2
Ib	C_3_H_7_	C_2_H_5_	$\varepsilon_{⊥}\;$(1 kHz) $\varepsilon_{∥}$ (1 kHz)	3.17 3.66
Ic	C_5_H_11_	CH_3_	∆ε (1 kHz)	0.49
Id	C_5_H_11_	C_2_H_5_	*n*_o_ (589 nm)	1.4507
	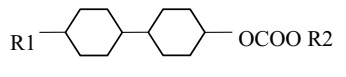		*n*_e_ (589 nm)	1.5062
II	C_3_H_7_	CH_2_CF_3_	∆n (589 nm)	0.056
	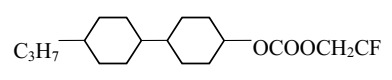			
III	C_3_H_7_	C_3_H_7_